# Effects of low carbohydrate diet compared to low fat diet on reversing the metabolic syndrome, using NCEP ATP III criteria: a randomized clinical trial

**DOI:** 10.1186/s40795-021-00466-8

**Published:** 2021-11-03

**Authors:** Sherzad Ali Ismael

**Affiliations:** Ass. Professor of Community Medicine, Kurdistan Board of Medical Specialties, Erbil, Iraq

**Keywords:** Metabolic syndrome, Reversing, Low carbohydrate diet, Low fat diet, NCEP ATP III, Erbil, Iraq

## Abstract

**Introduction:**

The purpose of this study is to compare the change in the metabolic syndrome prevalence and risk factors between participants who followed a low carbohydrate diet and those who followed a low fat diet for six months in Erbil city/ Iraqi Kurdistan.

**Methods:**

Out of 289 apparently healthy obese adults who were chosen by a stratified multistage probability sampling method, 94 of them agreed to participate in the study. They were assigned to low carbohydrate and low fat diet groups. Both groups were followed up for 6 months and the data were taken at baseline, after 3 months and after 6 months of intervention. Ninety-four obese adults completed the intervention. One-way repeated measures ANOVA was used to compare differences of metabolic dependent variables between the two independent variables, the low carbohydrate and low fat diet, at baseline, after 3 months and after 6 months of intervention.

**Results:**

The Participants in low carbohydrate diet group had greater decrease in the prevalence of MetS. At the baseline, according to the ATP III criteria, the prevalence of metabolic syndrome was 44.4% (24/54) in low carbohydrate diet group and 60% (24/40) in low fat diet group. The prevalence of MetS was decreased significantly to 16.7% (9/54) after 3 months and to 3.7% (2/54) after 6 months in low carbohydrate diet (*p* < 0.001). Moreover, the prevalence of MetS was decreased significantly to 32.5 (13/40) after 3 months and to 22.5% (9/40) after 6 months in low fat diet (*p* < 0.001). No statistically significant difference was found between low carbohydrate diet & low fat diet at the baseline (*p*-value = 0.136) and after 3 months and after 6 months of intervention.

**Conclusions:**

Both low carbohydrate diet and low fat diet have significant effects on reducing the prevalence of MetS in obese adults when followed up for 6 months. Compared to low fat diet, low carbohydrate diet had greater effect in reducing the prevalence of metabolic syndrome. Both diet programs were found to be effective in improving the metabolic state of obese adults.

**Trial registration:**

The trial is registered retrospectively at the US National Institutes of Health (ClinicalTrials.gov). The registration in the US National Institutes of Health was done in 23/12/2020 with the registration number: NCT04681924.

## Introduction

The Metabolic Syndrome (MetS) is a leading and raising public health and clinical concern worldwide due to many factors including urbanization, excess calorie intake, increasing obesity trends as well as sedentary life style. Metabolic syndrome has a major effect on increasing the risk of type 2 diabetes mellitus (T2DM) and the risk of developing cardiovascular disease (CVD) in the next 10 years [1]. The prevalence of MetS globally ranges from < 10% to as much as 84%, varies according to the region, urban or rural environment, population composition (such as population demography), and the definition of the syndrome used [2]. The NCEP ATP III definition is adopted as one of the most widely used criteria for diagnosing metabolic syndrome. It combines the key features of hyperglycaemia/ insulin resistance, visceral obesity, dyslipidaemia and hypertension [3].

The treatment of metabolic syndrome usually aims to improve insulin sensitivity and treat the associated metabolic abnormalities [4]. In the past few years, studies assessed the health effects of low carbohydrate diet (LCD), versus low fat diet (LFD) [4, 5]. Low carbohydrate diet has become a widespread strategy for weight reduction and weight management in recent years [5]. In addition, data from several randomized trials showed that LCD produced greater effects on weight reduction and decreasing cardiovascular and metabolic risk factors than LFD [4–9]. However, the macronutrient ratios of low carbohydrate diets are not standardized. For instance, American Diabetes Association (ADA) recommends a low carbohydrate diet which restricted intake of carbohydrate to < 130 g/ day or 30 to < 40% of calories [10–14].

Although the pathogenesis of MetS is strongly linked to excessive food consumption, in particular fat intake, there is no consensus about the effects of LCD versus LFD on reversing the MetS and on its’ metabolic risk factors. Concerns have been raised with regard to the macronutrient shift with high CHO restriction and the substantial intakes of fats, which may unfavorably affect CVD risk factors [11–13]. Meanwhile the LFD has generally been supported to have beneficial effects on these risk factors [14, 15].

The purpose of this study is to compare the change in the metabolic syndrome prevalence and risk factors between participants who followed a low carbohydrate diet and those who followed a low fat diet for six months in Erbil city/ Iraqi Kurdistan. Specifically, the study attempts to find out estimation of the prevalence of MetS with their metabolic risk factors, identification of the effects of LCD & LFD on prevalence of MetS and on the metabolic outcomes.

## Methods

### Study design and study population

A Randomized multi-stage cluster sampling survey of the houses of Erbil city was used to determine the prevalence of MetS in Erbil city. Seven hundred healthy apparent adults (≥18 year old) (358 males and 442 females) were surveyed in the 12 population clusters in Erbil city. A non-randomized clinical trial conducted over 6 months, between (January and June 2017) with outcome assessments at baseline, after 3 months and after 6 months of intervention. Out of 289 obese adult participants who met the inclusion criteria to participate in the trial, only 94 of them completed the 6 months of intervention (23 males and 71 females). They were non-randomly assigned to two groups, the LCD (*n* = 54) and LFD (*n* = 40) group. All participants completed comprehensive medical examination and routine blood tests at baseline, after 3 months and after 6 months of intervention.

The trial was retrospectively registered at the US National Institutes of Health (ClinicalTrials.gov) in 23/12/2020 with the registration number: NCT04681924.

A modified questionnaire of world health organization (WHO) STEPwise approach to Surveillance of noncommunicable diseases (STEPS) was used in this study. The modified questionnaire included 25 questions on socio-demographic (9 questions), anthropometric measurements (7 questions) and biochemical measurements (9 questions) [16].

According to the NCEP ATP III guidelines, in the current study, the participants are diagnosed as having Metabolic Syndrome if they possess three or more of the following criteria illustrated in (Table 1).

**Table 1:** The ATP III components and criteria of diagnosing metabolic syndrome [3]

Component	Criteria
Abdominal obesity: Increased waist circumference	Men: ≥ 102 cm Women: ≥ 88 cm
Elevated triglycerides	≥ 150 mg/dL
Reduced HDL	Men: < 40 mg/dL Women: < 50 mg/dL
Elevated blood pressure	≥ 130/85 mmHg
Elevated fasting glucose	≥ 100 mg/dL

Individuals with history or diagnosed with diseases and health related issues like diabetes mellitus, hypertension, chronic skin disease, heart disease, hyperlipidemia, malignant disease, and rheumatoid arthritis and those who had undergone surgery during one month before the study, were excluded in the study.

### Diet composition

The target macronutrient composition of a low carbohydrate diet was a diet allowing an intake of carbohydrate to < 130 g/ day or 30 to < 40% of calories per day without energy intake restriction [10]. While the target macronutrient composition of a low fat diet allows a maximum of 20–35% of the daily energy intake from fat [14]. Written meal plans and details instructions of both low carbohydrate and low fat diet was given to the participants in their native language (Kurdish). All participants were provided with journals, recipe ideas, information on how to keep accurate food records, and detailed food composition lists to assist with compliance. The food consumption was tracked by food log designed by the researcher. The participants met the researcher in weekly basis for weight measurements and diet consultation.

### Study intervention

More than half of the participants (*n* = 54) were followed the LCD. In this diet program, the primary behavioral target of LCD was to limit carbohydrate intake. Therefore, the limited carbohydrate intake and unrestricted consumption of fat and protein were allowed. During the first two weeks of the intervention, participants were instructed to limit carbohydrate intake to < 130 g per day.

Forty participants were followed the LFD. The primary behavioral target of LFD was to limit the overall energy intake (1200 kcal/d). They received instructions to increase calorie intake from 1200 to 1800 kcal per day (≤ 30% of calories from fat, < 7% saturated fat) [7–9]. The adherence of the participants to the diet programs was recorded based on participants’ self-reporting. Delivery of the intervention for both groups was not blinded. The investigator generated the random allocation sequence, enrolled participants, and assigned participants to the interventions.

### Study outcomes

The primary outcomes of the study were: Prevalence of metabolic syndrome, change in the prevalence of metabolic syndrome from baseline and comparing it in both diet programs at baseline, after 3 months and after 6 months of intervention. Regarding the prevalence of metabolic syndrome, the number of participants in the sample with the criteria of metabolic syndrome, divided by the total number of participants in the sample. According to the NCEP ATP III guidelines, the participants diagnosed with metabolic syndrome if they possess three or more of the following criteria: Abdominal obesity, elevated triglycerides, reduced HDL, elevated blood pressure and elevated fasting glucose.

All 94 participants were followed up regularly by face-to-face interview every two weeks by the researcher. Each follow-up session lasted from 15 to 30 min. All the participants were prescribed the same level of instruction concerning drinking 10–12 glasses of water /day and physical activity (principally walking), beginning at week 2, with 1 session of 30–45 min per day then increasing the duration of physical activity to reach at least 60 min /day [8, 9].

The secondary outcomes of the study were: Body mass index (BMI), change from baseline in abdominal obesity, change from baseline in elevated triglycerides, change from baseline in reduced HDL, change from baseline in elevated blood pressure and change from baseline in elevated fasting glucose after 3 months and after 6 months of intervention.

An electronic weight scale (model 770: Seca, Germany) was used to measure the participants’ weight to the nearest 0.1 kg. While the participants were asked to stand still without shoes, a measuring tape was utilized to measure their lightly clothed height to the nearest 0.5 cm. The formula used for calculating BMI was as following: BMI = weight(kg)/height(m^2^) [16]. The measurements were collected at baseline, after 3 months and after 6 months.

Waist circumference (WC) was measured midway above umbilical between the distal border of the lowest rib and the superior border of the iliac crest at the end of a normal expiration. Measurements were done with the participant in upright position, without clothes, both feet touching the ground, and arms hanging freely. A non-elastic tape measure was placed directly on the skin on the waist line without putting pressure on the abdominal wall [17, 18].

Blood pressure (BP) was measured by the researcher using an MDF Desk Mercury Sphygmomanometer (*Model* No: *MDF* 800), with cuff sizes based on measured arm circumference. Blood pressure checked after participants were asked to sit and take rest for 10 min. The measurements were collected at baseline, after 3 and after 6 months.

Blood samples were obtained after participants fasted overnight (8–10 h). Blood samples were analyzed in the laboratory department of one of the two main public hospitals in Erbil city, the Rzgary Teaching Hospital. The serum analyzed by the same laboratory and by the same device (BIOTECNICA BT4500 Full Automated Chemistry Analyser, 2016).

### Sample size and data analysis

Population size of Erbil governorate (for finite population correction factor or fpc) (N): 1,500,000, hypothesized % frequency of outcome factor in the population (p):35%+/− 5, confidence limits as % of 100 (absolute +/− %) (d): 5% and the design effect (for cluster surveys-DEFF): 2. For the confidence level of 95%, the sample size for estimation of the prevalence of MetS was estimated to be 699, for the convenience of the sample, 700 healthy apparent adults was taken. Sample size was calculated by n = [DEFF*Np(1-p)]/ [(d2/Z21-α/2*(N-1) + p*(1-p)]. Results from OpenEpi, Version 3, open-source calculator—SSPropor.

Data on baseline characteristics of participants were expressed as means ± SD and/ or frequencies and percentage. The data were checked for normal distribution by Shapiro-Wilk test; the *p*-value was 0.10 which indicates that the data were normally distributed. The study used t-test and Chi square test of association to compare baseline characteristics between both diet programs. When the expected count more than 20% of the cells of the table was less than 5, Fisher exact test was used. One-way repeated measure ANOVA was used to compare differences of metabolic dependent variables by the time points, at baseline, after 3 months and after 6 months of intervention. To check whether our non-significant results between LCD and LFD groups were due to a lack of statistical power, we conducted post hoc power analyses using GPower computer program (Faul & Erdfelder, 1992) version 3.1.9.6 with power (1 - β) set at 0.89, medium effect size = 0.50 and α = 05, two-tailed. This shows that sample size would have to increase up to *N* = 169; LCD = 85 and LFD = 84, in order for group differences to reach statistical significance at the 0.05 level. Thus, it is unlikely that our negative findings can be attributed to a limited sample size. A *p*-value of ≤0.05 was considered as the level of significance for all analyses. The Statistical Package for the Social Sciences (SPSS) software, version 22 was used for data analysis.

## Results

**Fig. 1:**
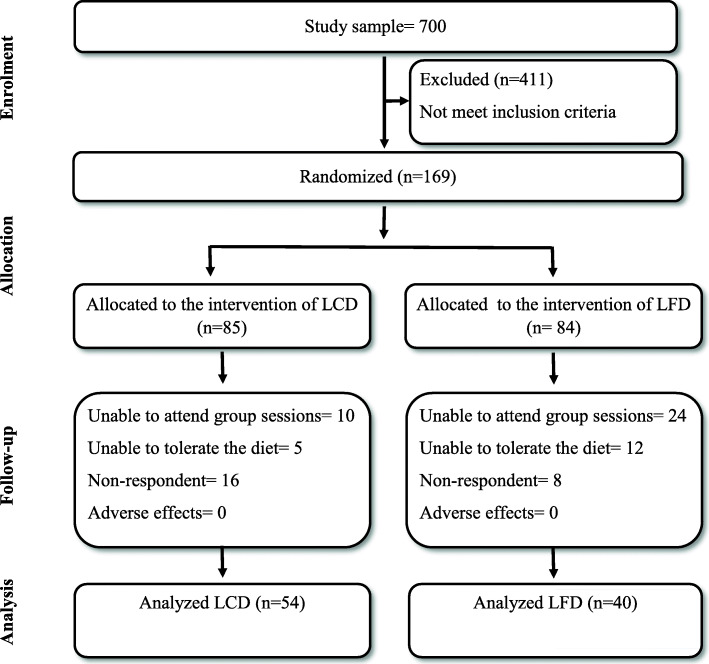
Flow diagram of the progress through the phases of a parallel randomized trial of two groups

In this study, 700 apparently healthy adult participant were recruited for the study; 411 of them did not met the inclusion criteria, versus 289. Out of those 289 obese participants, 169 of them agreed to participate in the intervention program. They are assigned non-randomly to either LCD or LFD groups. Among those participated in the intervention, 34 of them were unable to attend the group sessions, 17 of them were unable to tolerate the diet programs and 24 of them were regarded as non-respondents. The details of the participants who were randomly assigned and completed the period of intervention of each diet program are illustrated in Fig. 1.

Both LCD group 54 (57.4%) and LFD 40(42.6%) group of adults were completed the 6 months of dietary intervention. No statistically significant differences of the gender, ages and anthropometric measures at the baseline were found between LCD and LFD (*p* > 0.05). The descriptive statistics of the gender, age and anthropometric measurements of participants of both groups at the baseline are shown in the Table 2.

**Table 2:** Baseline characteristics of adult obese participants (*N* = 94)

Characteristics	LCD (***n*** = 54)		LFD (***n*** = 40)	
Male, n (%)	12	52.2%	11	47.8%
Female, n (%)	42	59.2%	29	40.8%
Mean age ± SD (years old)	39.02	9.74	40.17	9.09
Mean height ± SD (cm)	160.56	8.52	160.90	8.79
Mean weight ± SD (kg)	90.06	10.93	89.16	12.28
Mean body mass index ± SD (kg/m^2^)	34.96	3.64	34.42	3.85
Mean waist circumference ± SD (cm)	106.67	8.58	106.50	10.52
Mean hip circumference ± SD (cm)	113.24	8.22	111.68	10.95
Mean triglycerides ± SD (mg/dl)	127.79	73.77	161.34	119.59
Mean high density lipoproteins cholesterol (mg/dl)	41.47	6.74	42.11	9.06
Mean systolic blood pressure (mm Hg)	122.50	15.50	123.38	14.16
Mean diastolic blood pressure (mm Hg)	80.00	9.76	82.25	13.35
Mean fasting blood glucose (mg/dl)	0.15	0.36	0.20	0.41

**Fig. 2:**
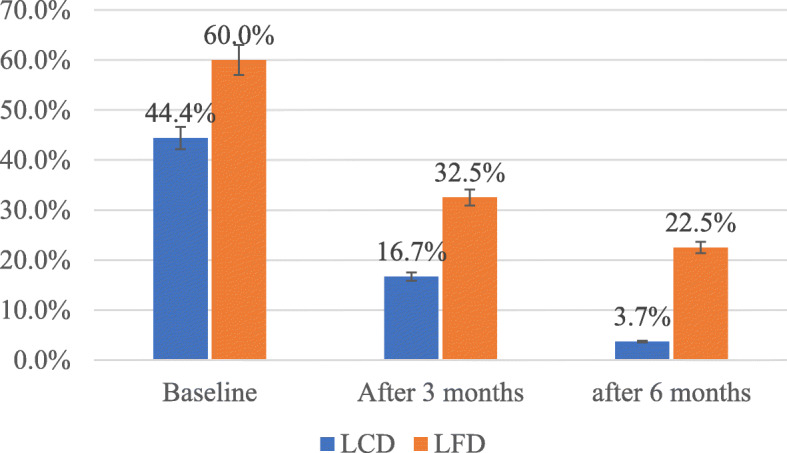
Prevalence of MetS from baseline, after 3 months and after 6 months of intervention in LCD & LFD groups. Standard error of the bars representing the prevalence of MetS

Figure 2 shows the prevalence of metabolic syndrome among adults at the baseline, after 3 months and after 6 months of intervention. At the baseline, according to the ATP III criteria, the prevalence of metabolic syndrome was 44.4% (24/54) in LCD group and 60% (24/40) in LFD group. No statistically significant difference was found between LCD & LFD at the baseline (*p*-value = 0.136), after 3 months and after 6 months of intervention. The prevalence of MetS decreased significantly to 16.7% (9/54) after 3 months and 3.7% (2/54) after 6 months in LCD (*p* < 0.001). Moreover, the prevalence of MetS was decreased significantly to 32.5 (13/40) after 3 months and 22.5% (9/40) after 6 months in LFD (*p* < 0.001).

Table 2 shows the results of a repeated measures ANOVA with both a Greenhouse-Geisser correction of WC, TG, HDL, DBP and FGL and a Sphericity Assumed correction of SBP. The mean of WC differed statistically not significantly between time points (baseline, after 3 months & after 6 months) (F (1.00, 93.00) = 4.13, *p* = 0.135). Post hoc tests using the Bonferroni correction revealed that intervention elicited no changes in WC from baseline and after 3 months of intervention (0.97 ± 0.14). However, WC had been decreased to 0.93 ± 0.24 after 6 months, but also was statistically not significantly different to the baseline and after 3 months of intervention (*p* = 0.135).

The mean TG was differed statistically significantly between time points (baseline, after 3 months & after 6 months) (F (1.58, 147.39) = 21.08, *p* < 0.001). Post hoc tests using the Bonferroni correction revealed that intervention elicited decrease in TG from baseline and after 3 months of intervention (0.31 ± 0.46 vs 0.14 ± 0.35), which was statistically significant (*p* < 0.001). Also, mean TG had been decreased to 0.07 ± 0.26 after 6 months, which was statistically significantly different to the baseline (*p* < 0.001) and after 3 months of intervention (*p* = 0.022).

The mean HDL was differed statistically significantly between time points (baseline, after 3 months & after 6 months) (F (1.62, 150.70) = 47.13, *p* < 0.001). Post hoc tests using the Bonferroni correction revealed that intervention elicited decrease in HDL from baseline and after 3 months of intervention (0.78 ± 0.41 vs 0.39 ± 0.49), which was statistically significant (*p* < 0.001). Also, mean HDL had been decreased to 0.27 ± 0.44 after 6 months, which was statistically significantly different to the baseline (*p* < 0.001) and after 3 months of intervention (*p* = 0.012).

The mean SBP differed statistically significantly between time points (baseline, after 3 months & after 6 months) (F (2, 186) = 3.34, *p* = 0.037). Post hoc tests using the Bonferroni correction revealed that intervention elicited a slight decrease in SBP from baseline and after 3 months of intervention (0.24 ± 0.43 vs 0.10 ± 0.30), which was statistically significant (*p* = 0.032). However, the mean SBP had been increased to 0.15 ± 0.36 after 6 months, which was statistically not significantly different to the baseline (*p* = 1.000) and after 3 months of intervention (*p* = 0.287).

Furthermore, the mean DBP differed statistically significantly between time points (baseline, after 3 months & after 6 months) (F (1.72, 160.60) = 11.54, *p* < 0.001). Post hoc tests using the Bonferroni correction revealed that intervention elicited decrease in DBP from baseline and after 3 months of intervention (0.24 ± 0.43 vs 0.12 ± 0.33), which was statistically not significant (*p* = 0.081). However, the mean DBP decreased to 0.03 ± 0.17 after 6 months, which was statistically significantly different to the baseline (*p* < 0.001) and after 3 months of intervention (*p* = 0.035).

Finally, the mean FGL differed statistically significantly between time points (baseline, after 3 months & after 6 months) (F (1.82, 169.56) = 4.85, *p* = 0.011). Post hoc tests using the Bonferroni correction revealed that intervention elicited decrease in FGL from baseline and after 3 months of intervention (0.21 ± 0.41 vs 0.19 ± 0.39), which was not statistically significant (*p* = 1.00). However, the mean FGL decreased to 0.10 ± 0.30 after 6 months, which was statistically significantly different to baseline (*p* = 0.035) and after 3 months of intervention (*p* = 0.032).

Regarding the mean changes of metabolic risk factors between LCD and LFD groups, it was found that the mean change ± SD of only TG was statistically significant after 3 months and after 6 months of intervention; levels of TG decreased significantly in both groups, with greater decrease among participants in the LFD at 3 months and at 6 months of intervention (*p* = 0.041 and *p* = 0.012), respectively. All other changes of metabolic risk factors between LCD and LFD were not statistically significant at 3 and 6 months of interventions (*p* > 0.05), Table 3.

**Table 3:** Main effects of the intervention of diet programs on MetS components between time points (baseline, after 3 months and after 6 months)

Anthropometric measures		Mean ± SD	95% Confidence Interval for Mean
WC	Baseline	0.97 ± 0.14	(0.94, 1.00)
	After 3 months	0.97 ± 0.14	(0.94, 1.00)
	After 6 months	0.93 ± 0.24	(0.88, 0.98)
TG	Baseline	0.31 ± 0.46	(0.22, 0.41)
	After 3 months	0.14 ± 0.35	(0.07, 0.22)
	After 6 months	0.07 ± 0.26	(0.02, 0.12)
HDL	Baseline	0.78 ± 0.41	(0.70, 0.87)
	After 3 months	0.39 ± 0.49	(0.29, 0.49)
	After 6 months	0.27 ± 0.44	(0.18, 0.36)
SBP	Baseline	0.19 ± 0.39	(0.11, 0.27)
	After 3 months	0.10 ± 0.30	(0.04, 0.17)
	After 6 months	0.15 ± 0.36	(0.08, 0.23)
DBP	Baseline	0.24 ± 0.43	(0.15, 0.33)
	After 3 months	0.12 ± 0.33	(0.05, 0.19)
	After 6 months	0.03 ± 0.17	(−0.04, 0.06)
FGl	Baseline	0.21 ± 0.41	(0.12, 0.29)
	After 3 months	0.19 ± 0.39	(0.11, 0.27)
	After 6 months	0.10 ± 0.30	(0.04, 0.17)

Abbreviations: WC, waist circumference; TG, total glycerides; HDL, high density lipoprotein; SBP, systolic blood pressure; DBP, diastolic blood pressure; FGl, fasting glucose

## Discussion

There is a growing evidence supporting use of low carbohydrate diet and its association with a decreased risk of MetS and its components in adults [19]. The purpose of this study was to compare the changes in the prevalence of metabolic syndrome and its risk factors among obese adults who followed a low carbohydrate diet and those who followed a low fat diet for three and six months. In the present study, the effects of LCD and LFD programs on reducing the prevalence of MetS were compared and its risk factors were scrutinized. It was found that both LCD and LFD dietary programs were effective in reducing the prevalence of MetS after 3 months and after 6 months of intervention. Obese adults on LCD had a greater decrease in the prevalence of MetS than LFD participants.

The findings of the current study were similar to the findings of many studies. A study that following up the participants for 12 months, found that both a very low carbohydrate, high saturated fat diet and a high carbohydrate, low fat diet resulted in similar weight loss and changes in body composition. The LC diet may offer clinical benefits to obese persons with insulin resistance [20]. Similarly, a study aimed to evaluate the effects of 2-year treatment with a low-carbohydrate or low-fat diet, each of which was combined with a comprehensive lifestyle modification program, found that the weight loss can be successfully achieved with either a low fat or low carbohydrate diet when tailored with behavioral treatment [21].

Although this study showed that both diet programs were effective in improving the metabolic state of participants’ WC, TG, HDL, DBP and FGl, and no significant difference was found between LCD & LFD on these metabolic risk factors except on TG. The LFD was found to have greater effect on decreasing the TG than LCD. As explained earlier, the non-significant results between LCD and LFD groups were not due to a lack of statistical power, that showed by using GPower computer program (Faul & Erdfelder, 1992) version 3.1.9.6 with power (1 - β) set at 0.89, medium effect size = 0.50 and α = 05, two-tailed. Thus, it is unlikely that the negative findings between LCD & LFD might be attributed to a limited sample size. Furthermore, still there is no consensus about the effects of LCD versus LFD on reducing the prevalence of MetS and on its metabolic risk factors [10–13]. However, concerns have been raised with regard to the macronutrient shift with high CHO restriction and the substantial intakes of fats, which may present unfavorable effects on CVD risk factors [11–13]. Meanwhile the LFD has generally been supported by studies to have beneficial effects on these risk factors [14, 15].

In addition, several diet comparisons have been published showing that LCD was at least as effective as LFD on weight loss, lipid profile, and other health markers [6, 18–21]. Meanwhile a randomized controlled trial evaluating the effects of LCD v. LFD on weight loss and risk factors of CVD; participants on LCD experienced a larger reduction in body weight (weighted mean difference − 2.17 kg; 95% CI –3.36, − 0.99) and TG (weighted mean difference − 0.26 mmol/l; 95% CI –0.37, − 0.15), a greater increase in HDL-cholesterol (weighted mean difference 0.14 mmol/l; 95% CI 0.09, 0.19) and decrease in LDL-cholesterol (weighted mean difference 0.16 mmol/l; 95% CI 0.003, 0.33) [19]. Likewise, weighted mean difference for SBP, DBP and glucose and insulin levels between the LCD and LFD groups were not significant [12].

Most calorie-decreasing diets cause clinically important weight loss as long as the diet is continued as the findings supported by recent recommendations for weight loss [19]. Decreasing consumption of dietary carbohydrates and changing it with either fat or protein has been shown to reduce TG and increase HDL cholesterol even under weight-stable circumstances [20]. The increased HDL cholesterol during the LCD will result in the increased fat consumption [20]. Therefore, studies suggest that lowering TG levels has an overall cardiovascular benefit (23).

In most of the dietary intervention, it is very difficult to anticipate 100% dietary adherence, because of a dietary intervention on free-living subjects. If food and energy conditions were carefully controlled in dietary interventions, reporting the food intake by participant themselves is still a limitation. In addition, people may also forget to report certain foods or they report what is anticipated rather than their actual food intake [12]. This remains one of the main limitations for all dietary interventions including the current study.

Clearly, all of these diets have benefits but they can be realized only when they are followed. However, a common concern of all dietary intervention studies including the current study is poor long-term adherence [14]. Furthermore, collection diet related data among the studies, and methods of dietary assessment have limitations. Such limitations may include measurement errors, for example under-or over-reporting of certain types of foods. Finally, inability to differentiate between the effects of diet, exercise, and weight is also regarded as another limitation of the study. Nevertheless, this study supports the concept and approach of reversing metabolic syndrome and its associated risk factors by dietary intervention, especially the LCD.

## Conclusions

Both LCD and LFD have significant effects on reducing the prevalence of MetS of obese adults when followed up for 6 months. Low carbohydrate diet had greater effect on reducing the prevalence of metabolic syndrome compared to LFD. Both diet programs were effective in improving the metabolic state of obese adults’ WC, TG, HDL, DBP and FGl after 6 months with no significant difference between LCD & LFD on these metabolic risk factors except on TG. The LFD was found to have greater effect on decreasing the TG than LCD.

## Data Availability

The datasets used and analyzed during the current study are available from the corresponding author on reasonable request. The blood samples are stored at the center laboratory of Erbil until the end of the study then the specimen containers were discarded into special disinfectant-filled containers.

## Abbreviations

MetS: Metabolic Syndrome
LCD: Low Carb Diet
LFD: Low Fat Diet
NCEP: National Cholesterol Education Program
ATP: Adult Treatment Panel III
TG: Triglyceride
HDL: High Density Lipoprotein
LDL: Low Density Lipoprotein
CVD: Cardio Vascular Disease
CM: Centimeter
DM: Diabetes Mellitus
BP: Blood Pressure
WC: Waist Circumference
